# Hypomethylating Agents for Treatment of Elderly Patients with Refractory Acute Myeloid Leukemia - A Case Report with a Focused Review of Literature

**DOI:** 10.7759/cureus.2734

**Published:** 2018-06-05

**Authors:** Pavan Tenneti, Govardhanan Nagaiah

**Affiliations:** 1 Department of Medicine, Banner University Medical Center Tucson, Tucson, USA; 2 Oncology, Arizona Oncology Associates, PHOENIX, USA

**Keywords:** azacytidine, decitabine, refractory, aml, elderly

## Abstract

The prognosis of elderly patients with acute myeloid leukemia (AML) is poor. Intensive chemotherapy with the combination of cytarabine and anthracyclines is typically used as the first-line treatment in the elderly with newly diagnosed AML who are able to tolerate this regimen. Unfortunately, many patients are refractory to this treatment approach. The role of hypomethylating agents in the treatment of elderly patients with refractory AML has not been clearly defined. Therefore, we conducted a focused literature review to assess the role of hypomethylating agents in elderly patients with refractory AML.

In addition, we present a case report of a patient with refractory AML, who was subsequently treated with azacytidine and showed an immediate response after one treatment cycle. He then proceeded to undergo nine more cycles. Ten months after the start of treatment with azacytidine, he remains in complete remission with incomplete hematologic recovery. Given the positive results noted in multiple retrospective studies and in the presented case report, large-scale, prospective studies are needed to further define the role of hypomethylating agents in the treatment of elderly patients with refractory AML.

## Introduction

The prognosis of elderly patients with acute myeloid leukemia (AML) is poor. The risk of adverse prognostic factors, including unfavorable cytogenetics, secondary AML, and treatment-associated AML, increase with age [1-4]. Intensive chemotherapy in the elderly with the combination of cytarabine and anthracycline (7 + 3 regimen) has resulted in a complete response (CR) or complete response with incomplete hematological recovery (CRi) in 45% – 64% patients, with an overall survival (OS) of 26% – 31% at one to two years [5-6]. In a 2006 study by Kantarjain et al., in patients with more than three adverse factors (i.e., age > 75 years, complex karyotype, performance status > 2, prior history of myelodysplastic syndrome, and creatinine level > 1.3 mg/dl), the reported CR/CRi was 19%, the eight-week induction mortality was 65%, and the median overall survival (MOS) was one month. In contrast, in the same study, in patients with zero to one adverse factor, the reported CR/CRi was 69%, the eight-week induction mortality was 10%, and the MOS was 16 months [5]. These studies demonstrate that many elderly patients, particularly those with poor baseline prognostic factors, are refractory to induction treatment with intensive chemotherapy.

There is no standard of care therapy for elderly patients with refractory AML. Hypomethylating agents, including azacytidine (AZA) and decitabine, can be used as induction treatment in patients with newly diagnosed AML who are not candidates for intensive chemotherapy, have poor performance status, or have adverse cytogenetics [7-8]. The role of these drugs in elderly patients with refractory AML has not been clearly established. Herein, we report the findings of a literature review to define the role of hypomethylating agents in elderly patients with refractory AML and present a case report of a patient with refractory AML treated with AZA.

## Case presentation

**Figure 1:**
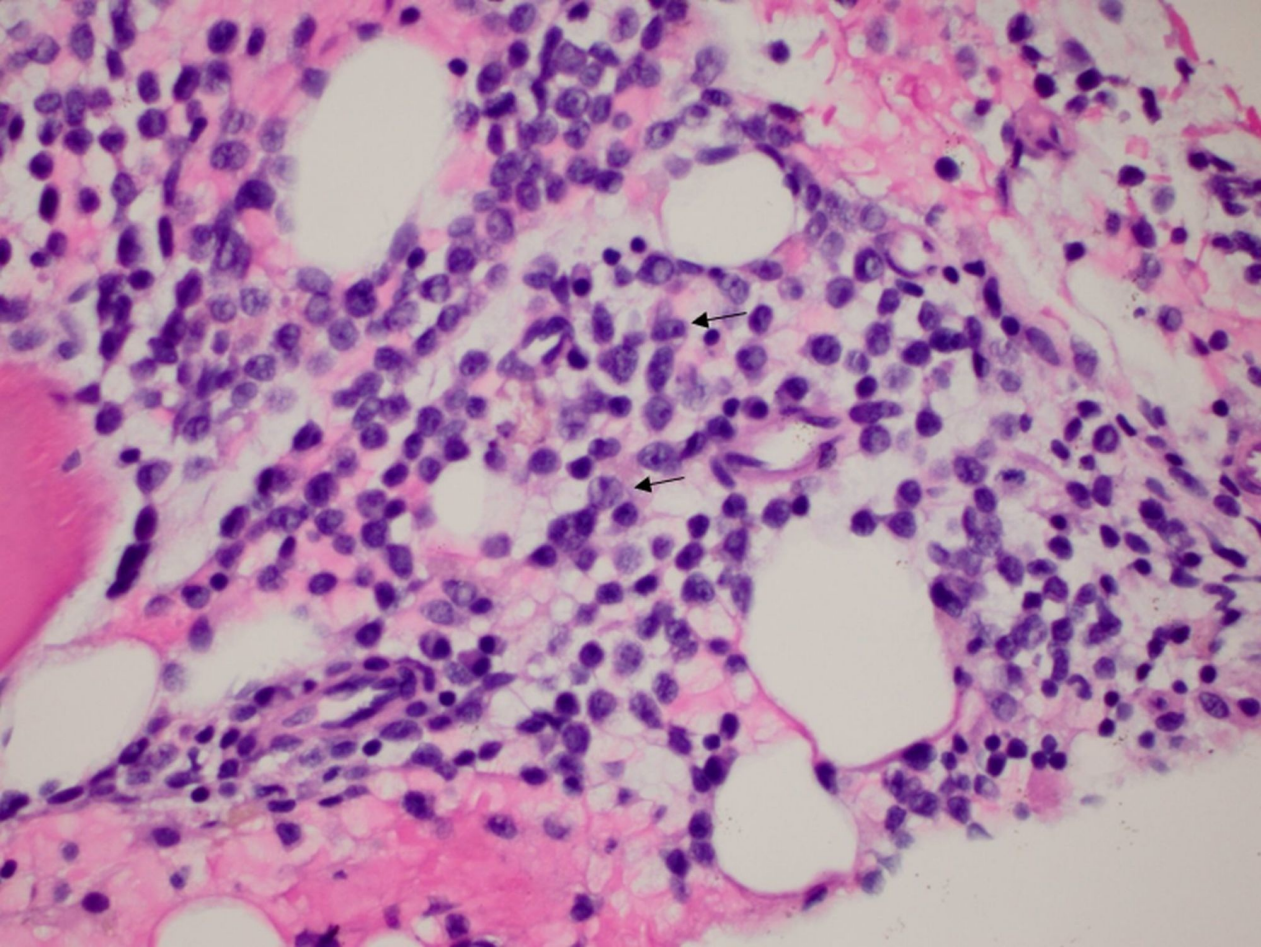
Acute myeloid leukemia with 70% blasts in bone marrrow

**Figure 2:**
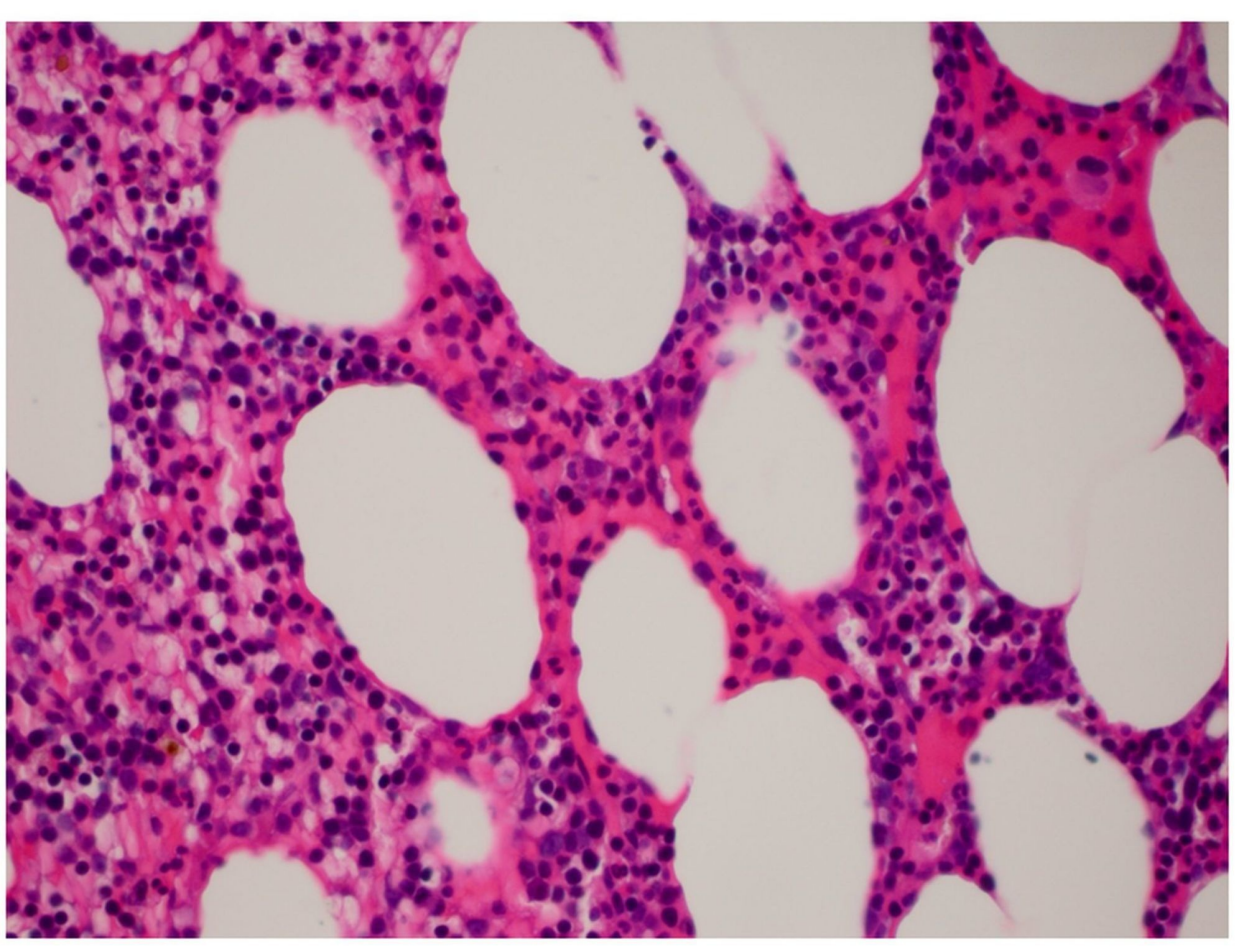
Hypercellular bone marrow with no blast cells

We present the case of a 76-year-old male with history of hypertension and deep vein thrombosis. He initially presented to the oncology clinic in June 2016 with a low white blood cell (WBC) (2,700 cells/microliter) and platelet counts (58,000 cells/microliter), which was found during routine blood work. Initial bone marrow biopsy performed in June 2016 showed normocellular marrow with no evidence of blasts. Fluorescence in situ hybridization (FISH) did not show evidence of myelodysplastic syndrome (MDS). The patient was treated conservatively and was given a trial of steroids. He did not respond to these treatments, and blood tests performed in February of 2017 showed a platelet count of 39,000 cells/microliter, a hemoglobin level of 7.8 gm/dl, and a WBC count of 2,000 cells/microliter. In view of the persistent trilineage depressed blood counts, a second bone marrow biopsy was performed in March of 2017, which revealed 20.8% blasts with hypercellular bone marrow. Therefore, he was diagnosed with AML. He underwent additional cytogenetic testing, which showed that he did not have any of the favorable cytogenetics, including mutations of the CCAAT/enhancer-binding protein alpha (CEBPA) gene or nucleophosmin (NPM) 1 gene. Unfavorable FMS-like tyrosine kinase 3/internal tandem duplication (FLT3/ITD) mutation was detected with a polymerase chain reaction (PCR) product of 327 base pairs (bp). In addition, another poor prognostic marker, trisomy of the 21st chromosome, was detected. We administered the standard first-line induction chemotherapy regimen to the patient, including cytarabine (100 mg/m^2^) and daunorubicin (60 mg/m^2^), in March 2017. Unfortunately, a bone marrow biopsy performed 14 days after chemotherapy initiation showed AML with 72% blasts (Figure 1). At that time, the patient decided that he did not want to continue with re-induction attempts involving intensive chemotherapy. After detailed discussions with the patient and a literature review, we presented the option of salvage chemotherapy with the less-toxic AZA for the treatment of his refractory AML. He agreed to this treatment, and we administered AZA (75 mg/m^2^) for seven days in the second half of March 2017. A subsequent bone marrow biopsy performed in April 2017 showed a positive response with no morphologic or immunophenotypic evidence of increased blasts. Subsequently, he received nine more cycles of AZA, which he tolerated very well. A repeat bone marrow biopsy performed in January 2018 revealed no blasts (Figure 2). The patient continues to be in complete remission with incomplete hematologic recovery (low platelet counts - 44000 cells/microliter) after 10 cycles of treatment with AZA.

## Discussion

Intensive chemotherapy, with a combination of cytarabine and anthracycline, remains the preferred induction treatment in elderly patients with newly diagnosed AML who can tolerate the medications. Kantarjian et al. (2006) reviewed 798 patients and reported a CR/CRi of 45%, MOS of 20 weeks, and OS of 27% at one-year [5]. In a 2009 study, Lowenberg et al. compared the outcomes of elderly patients who received high doses of daunorubicin (90 mg) with those who received lower doses (45 mg) and found significantly higher complete response rates associated with the high dose (64% vs 54%, p = 0.02). However, at two-years, the long-term outcomes of event-free survival (EFS) (20% vs 17%) and OS (31% vs 26%) were not statistically different [6]. In 2017, Stone et al. reported that patients who received mitoxantrone in addition to standard induction chemotherapy had marginally superior CR/CRi (59% vs 54%) and four-year OS (51.4% vs 44.3%) compared to patients who received only cytarabine and daunorubicin [9]. Similarly, Castaigne et al. found that the addition of gemtuzumab ozogamicin to daunorubicin and cytarabine was associated with an improved two-year EFS (41% vs 17%, p = 0.0003) and OS (53% vs 42%, p = 0.03) compared to the use of the latter drugs alone [10]. In patients who cannot tolerate anthracyclines, clofarabine could be a potential alternative as induction chemotherapy, as determined by Kantarjain et al. (2010) [11]. They reported CR/CRi to be 46%, median disease-free survival to be 37 weeks, and MOS to be 42 weeks. To summarize, the combination of cytarabine and anthracycline has CR/CRi of 45%–64% and OS at one to two years of 26% - 31%. Mitoxantrone and gemtuzumab ozogamicin, when added to standard chemotherapy, resulted in improved CR/CRi and MOS. Despite this response with intensive chemotherapy, many elderly patients remain refractory to induction treatment (Table 1).

**Table 1:** Intensive Chemotherapy Regimens in Elderly Patients with Newly Diagnosed AML AML: acute myeloid leukemia; C: cytarabine; CR/CRi: complete response/complete response with incomplete hematological response; D: daunorubicin; DFS: disease-free survival; EFS: event-free survival; GO: gemtuzumab ozogamicin; M: mitoxantrone; mDFS: median disease-free survival; MOS: median overall survival; NR: not reported; OS: overall survival; y: years

Author and Year	Drug combination	No. of patients	Response CR/CRi	EFS/Median DFS	OS
Kantarjian et al. 2006 [5]	D+C	798	45%	NR	MOS = 20 weeks; 1 y = 27%
Lowenberg et al. 2009 [6]	C+ high D; C+ low D	C+ high D = 402; C+ low D = 411	C+ high D = 64%; C+ low D = 54% p = 0.002	2 y C+ high D = 20%; C+ low D = 17%	2 y C+ high D = 31%; C+ low D = 26%
Stone et al. 2017 [9]	D+C+M or D+C	D+C+M = 360; D+C = 357	D+C+M = 59%; D+C = 54%	NR	MOS D+C+M = 74.7 m, D+C = 25.6; MOS at 4 y; D+C+M = 51.4%; D+C = 44.3%
Castaigne et al. 2012 [10]	D+C+GO	280 D+C+GO = 140; D+C = 140	D+C+GO = 81%; D+C = 75%	2 y D+C+GO = 41%; D+C = 17% (p = 0.0003)	2 y D+C+GO = 53%; D+C = 42% (p = 0.03)
Kantarjain et al. 2010 [11]	Clofarabine	112	46%	mDFS = 37 weeks	MOS = 41 weeks

Epigenetic alterations, such as DNA methylation and histone modifications, are abnormal in cancer cells, and the use of the hypomethylating agents are important additions to the arsenal of active cancer drugs, particularly for the treatment of the myelodysplastic syndromes and AML [12]. The National Comprehensive Cancer Network recommends the use of hypomethylating agents in the treatment of elderly patients with newly diagnosed AML who are not candidates for intensive chemotherapy and in the treatment of secondary (with a background of hematologic bone marrow disease) therapy-related AML. In a phase III study by Fenaux et al. in 2010, 113 patients were assigned to receive either AZA (n = 55) or a conventional care regimen (best supportive case, low dose cytarabine, intensive chemotherapy; n = 58). They reported a CR/CRi of 18% and 16% with a MOS of 24.5 and 16 months in AZA and conventional groups, respectively [7]. In another phase III study in 2015, Dombret et al. randomized elderly patients to receive either AZA (n = 241) or best conventional care therapy (n = 247). They reported a CR/CRi of 27.8% and 25.1% with a MOS of 10.6 and 6.5 months in AZA and conventional groups, respectively [13]. In a phase III study by Kantarjian et al. (2012), 485 patients were assigned 1:1 to receive either decitabine or a conventional care regimen. They reported a CR/CRi of 17.8% and 7.8% (p = 0.01) with a MOS of 7.7 and 5  months in decitabine and conventional groups, respectively [8]. These results demonstrate that hypomethylating agents are a good alternative choice as the first line treatment option in elderly patients with newly diagnosed AML who are not candidates for intensive chemotherapy.

In elderly patients with refractory AML, subsequent lines of treatment are not clear. The role of hypomethylating agents in this patient population has not been clearly established. Through a literature review, we found seven studies where hypomethylating agents were used in elderly patients with refractory/relapsed AML. In a phase I study by Al-Ali et al. in 2012 (n = 20), patients with refractory/relapsed AML received AZA. None of the patients achieved CR/CRi, and the MOS was 2.9 months [14]. In addition, we found six other retrospective studies that used hypomethylating agents in patients with relapsed/refractory AML. In 2015, Gemueden et al. (n = 14) reported a MOS of 11 months in patients who received AZA [15]. Itzykson et al. studied the outcomes of patients with relapsed (n = 67) and refractory (n = 63) AML who received AZA [16]. They reported a CR/CRi of 17% for the whole group, response duration of 11.9 months in responders, and MOS of 8.4 months for entire study population (responders and non-responders) [16]. In another study from 2013 (n = 47), Ivanoff et al. reported a CR rate of 21% and a MOS of nine months [17]. Ram et al. (n = 34) reported a CR of 32%, a 1 - 1.5-year EFS of 10% - 33%, and an OS at one and two years of 16% and 54.5%, respectively. The reported MOS for patients with relapsed and refractory disease were nine and 16 months, respectively [18]. Two studies administered decitabine in patients with relapsed/refractory AML [19-20]. Khan et al. (n = 34) and Ritchie et al. (n = 42) reported CRs of 15.7 - 21% and MOS of 7 - 8.5 months. In addition, Khan et al. reported MOS separately for patients with secondary AML (12.4 months) and patients with intermediate (eight months) and adverse cytogenetics (10 months) [19]. To summarize, for most retrospective studies using AZA in patients with relapsed/refractory AML, the CR was 17% - 32% and MOS was 8.4 - 12.5 months. In studies using decitabine, the CR was 15.7% - 21% and MOS was 7 - 8.5 months. In a phase I study that used AZA, the long-term outcomes were poor with a MOS of 2.9 months only (Table 2).

**Table 2:** Hypomethylating Agents in Patients with Relapsed/Refractory AML AZA: azacytidine; CR/Cri: complete response/complete response with incomplete hematological recovery; d: days; EFS: event-free survival; HI: hematological improvement; Int: intermediate cytogenetics; m: months; MOS: median overall survival; NR: not reported; OS: overall survival; RD: response duration; ref: refractory; rel: relapsed; Retro: retrospective study; sAML: secondary acute myeloid leukemia; y: years

Author and Year	Type of study	Drug	No. of patients	Response	EFS	OS
Al-Ali et al. 2012 [14]	Phase I	AZA	20	HI = 10%, CR/CRI = 0%	NR	MOS = 2.9 m
Gemuenden et al. 2013 [15]	Retro	AZA	14	NR	NR	MOS = 11 m
Itzykson et al. 2015 [16]	Retro	AZA	Relapse = 67, Refractory = 63	17%	RD = 11.9 m	MOS = 8.4 m
Ivanoff et al. 2013 [17]	Retro	AZA	47	21%	NR	MOS = 9 m
Khan et al. 2017 [19]	Retro	Decitabine	34	21%	NR	MOS = 8.5 m, sAML = 12.4 m, Int = 8 m; Adverse = 10 m
Ram et al. 2017 [18]	Retro	AZA	34	32%	1 y = 33%, 1.5 y = 10%	1 y = 54.5%, 2 y = 16%, MOS rel = 9 m, MOS ref = 16 m
Ritchie et al. 2013 [20]	Retro	Decitabine	42	CR = 15.7%	NR	MOS = 209 d

Our patient was administered intensive chemotherapy with cytarabine and daunorubicin. He showed good performance status and no other significant medical problems, except for a history of hypertension and deep vein thrombosis. He did not have any favorable cytogenetics. He was positive for several adverse prognostic factors, including FLT3/ITD and trisomy of the 21st chromosome. He was refractory to first-line treatment with intensive chemotherapy. Based on the positive survival benefit noted with hypomethylating agents for refractory AML in multiple retrospective studies, we offered the same treatment to our patient. He showed an excellent response to the first cycle of therapy with AZA, with his bone marrow biopsy showing no increase in the number of blasts after the first cycle. Subsequently, he received nine more cycles of AZA. A repeat biopsy performed 10 months later showed that he continued to be in complete remission. The patient’s good baseline disease characteristics (except for two adverse cytogenetic factors) and no prior history of antecedent hematological bone marrow disease may have contributed to his good response to AZA.

## Conclusions

No standardized treatment has proven to be beneficial in the treatment of elderly patients with refractory AML. Our literature review, including multiple retrospective studies, showed improvement in survival outcomes when hypomethylating agents were used in the treatment of elderly patients with relapsed/refractory AML. Our patient responded well to salvage therapy with AZA, achieving a CR/CRi of 10 months after the start of therapy. The definitive role of hypomethylating agents in refractory AML needs further evaluation in large prospective randomized studies.

## References

[1] Appelbaum F, Gundacker H, Head D (2006). Age and acute myeloid leukemia. Blood.

[2] Takeyama K, Seto M, Uike N (2000). Therapy-related leukemia and myelodysplastic syndrome: a large-scale Japanese study of clinical and cytogenetic features as well as prognostic factors. Int J Hematol.

[3] Mohammadi M, Cao Y, Glimelius I (2015). The impact of comorbid disease history on all-cause and cancer-specific mortality in myeloid leukemia and myeloma - a Swedish population-based study. BMC Cancer.

[4] Leith C, Kopecky J, Godwin J (1997). Acute myeloid leukemia in the elderly: assessment of multidrug resistance (MDR1) and cytogenetics distinguishes biologic subgroups with remarkably distinct responses to standard chemotherapy. A Southwest Oncology Group study. Blood.

[5] Kantarjian H, O'Brien S, Cortes J (2006). Results of intensive chemotherapy in 998 patients age 65 years or older with acute myeloid leukemia or high-risk myelodysplastic syndrome: predictive prognostic models for outcome. Cancer.

[6] Löwenberg B, Ossenkoppele GJ, van Putten W (2009). High-dose daunorubicin in older patients with acute myeloid leukemia. N Engl J Med.

[7] Fenaux P, Mufti G, Hellström-Lindberg E (2010). Azacitidine prolongs overall survival compared with conventional care regimens in elderly patients with low bone marrow blast count acute myeloid leukemia. J Clin Oncol.

[8] Kantarjian H, Thomas X, Dmoszynska A (2012). Multicenter, randomized, open-label, phase III trial of decitabine versus patient choice, with physician advice, of either supportive care or low-dose cytarabine for the treatment of older patients with newly diagnosed acute myeloid leukemia. J Clin Oncol.

[9] Stone R, Mandrekar S, Sanford B (2017). Midostaurin plus chemotherapy for acute myeloid leukemia with a FLT3 mutation. N Engl J Med.

[10] Castaigne S, Pautas C, Terré C (2012). Effect of gemtuzumab ozogamicin on survival of adult patients with de-novo acute myeloid leukaemia (ALFA- 0701): a randomised, open-label, phase 3 study. Lancet.

[11] Kantarjian H, Erba H, Claxton D (2010). Phase II study of clofarabine monotherapy in previously untreated older adults with acute myeloid leukemia and unfavorable prognostic factors. J Clin Oncol.

[12] Kihslinger J, Godley L (2007). The use of hypomethylating agents in the treatment of hematologic malignancies. Leuk Lymphoma.

[13] Dombret H, Seymour J, Butrym A (2015). International phase 3 study of azacitidine vs conventional care regimens in older patients with newly diagnosed AML with >30% blasts. Blood.

[14] Al-Ali H, Jaekel N, Niederwieser D (2012). Azacitidine in patients with acute myeloid leukemia medically unfit for or resistant to chemotherapy: a multicenter phase I/II study. Leuk Lymphoma.

[15] Gemuenden C, Benz R, Senn O (2015). Efficacy of azacitidine in de novo and relapsed acute myeloid leukemia: a retrospective comparative study. Clin Lymphoma Myeloma Leuk.

[16] Itzykson R, Thepot S, Berthon C (2015). Azacitidine for the treatment of relapsed and refractory AML in older patients. Leuk Res.

[17] Ivanoff S, Gruson B, Chantepie S (2013). 5-azacytidine treatment for relapsed or refractory acute myeloid leukemia after intensive chemotherapy. Am J Hematol.

[18] Ram R, Gatt M, Merkel D (2017). Second line azacitidine for elderly or infirmed patients with acute myeloid leukemia (AML) not eligible for allogeneic hematopoietic cell transplantation - a retrospective national multicenter study. Ann Hematol.

[19] Khan N, Hantel A, Knoebel R (2017). Efficacy of single-agent decitabine in relapsed and refractory acute myeloid leukemia. Leuk Lymphoma.

[20] Ritchie E, Feldman E, Christos P (2013). Decitabine in patients with newly diagnosed and relapsed acute myeloid leukemia. Leuk Lymphoma.

